# Baseline unfolded protein response signaling adjusts the timing of the mammalian cell cycle

**DOI:** 10.1091/mbc.E23-11-0419

**Published:** 2024-05-20

**Authors:** Soham P. Chowdhury, Sabrina C. Solley, Elena Polishchuk, Julien Bacal, Julia E. Conrad, Brooke M. Gardner, Diego Acosta-Alvear, Francesca Zappa

**Affiliations:** a Department of Molecular, Cellular, and Developmental Biology, University of California, Santa Barbara, Santa Barbara, CA 93106; b Telethon Institute of Genetics and Medicine (TIGEM), 80078 Pozzuoli, Naples, Italy; c Altos Labs Bay Area Institute of Science, Altos Labs, Redwood City, CA 94065; Nanyang Technological University

## Abstract

The endoplasmic reticulum (ER) is a single-copy organelle that cannot be generated de novo, suggesting coordination between the mechanisms overseeing ER integrity and those controlling the cell cycle to maintain organelle inheritance. The Unfolded Protein Response (UPR) is a conserved signaling network that regulates ER homeostasis. Here, we show that pharmacological and genetic inhibition of the UPR sensors IRE1, ATF6, and PERK in unstressed cells delays the cell cycle, with PERK inhibition showing the most penetrant effect, which was associated with a slowdown of the G_1_-to-S/G_2_ transition. Treatment with the small molecule ISRIB to bypass the effects of PERK-dependent phosphorylation of the translation initiation factor eIF2α had no such effect, suggesting that cell cycle timing depends on PERK’s kinase activity but is independent of eIF2α phosphorylation. Using complementary light and electron microscopy and flow cytometry-based analyses, we also demonstrate that the ER enlarges before mitosis. Together, our results suggest coordination between UPR signaling and the cell cycle to maintain ER physiology during cell division.

## Abstract (executive summary)

Blocking the UPR sensors in unstressed cells affects cell cycle dynamics.

PERK has cell cycle roles that are independent of phosphorylating eIF2α.

The ER expands during the mammalian cell cycle.

## Abstract (executive summary)

The endoplasmic reticulum (ER) is a single-copy organelle that cannot be generated de novo, and its functions are controlled by the Unfolded Protein Response (UPR). How the UPR impacts the cell cycle remained unclear.

By investigating this connection, the authors describe distinct roles for each UPR sensor, with PERK activity significantly affecting cell cycle beyond its typical function in the Integrated Stress Response (ISR). They also show that the ER expands before mitosis, potentially linking ER expansion to partitioning during mitosis.

These findings highlight an unexplored UPR function in cell cycle regulation, potentially serving as a critical checkpoint for mitosis.

## INTRODUCTION

The mammalian cell cycle consists of four well-defined stages: Gap 1 (G_1_), Synthesis (S), Gap 2 (G_2_), and mitosis, that are coordinated through finely adjusted temporal control and cell-cycle checkpoints to ensure the correct inheritance of the genome and cellular components by the daughter cells. This coordination requires multiple signaling pathways to integrate information about genome and organelle integrity, and whether conditions for progression through the cell cycle are suitable (e.g., energy status and resource availability; Rhind and Russell, 2012; Barnum and O´Connell, 2014). Additionally, because cytokinesis requires cell growth, cells employ mechanisms to regulate their size and adjust cellular content to ensure correct organelle inheritance (Kuroiwa, 2010). This coordination of mechanisms is essential for organelle partitioning. While multicopy organelles such as mitochondria and peroxisomes tend to be partitioned symmetrically among daughter cells, some membrane-bound organelles, including the Golgi apparatus and the endoplasmic reticulum (ER), fragment during cell division, suggesting synchronization between organelle restructuring and the cell cycle (Thyberg and Moskalewski, 1998). Indeed, the partitioning of the Golgi apparatus during mitosis has been linked to the activity of the cell cycle kinases PKMYT1 and CDK1 (Nakajima *et al.*, 2008; Ayala and Colanzi, 2017; Huang *et al.*, 2022). Surprisingly, no such link has been described for the ER.

The ER is the largest organelle in the cell and the site of secreted and membrane protein biosynthesis, calcium storage, lipid synthesis, and endomembrane biogenesis (Voeltz and Prinz, 2007). The ER also possesses structural subdomains (e.g., sheets, tubules, ER exit sites, and ER-organelle membrane contact sites) and is distinctly organized in different cell types; while professional secretory cells show an enlarged rough ER, cells dedicated to lipid synthesis possess mostly tubular ER, reflecting their respective physiological roles (Shibata *et al.*, 2006, 2010). Moreover, because the ER is contiguous with the nuclear envelope, it maintains the separation of functions between the nucleoplasm and cytoplasm, otherwise topologically equivalent compartments (Dawson *et al.*, 2009; Walters *et al.*, 2012). The ER´s complexity has led to contested models explaining its structure and reorganization during mitosis, with some studies suggesting complete vesiculation and disassembly, while others indicate the formation of fenestrated mitotic ER sheets (Cotter *et al.*, 2007; Puhka *et al.*, 2007). Although the mechanisms behind ER inheritance remain unclear, it is agreed that the ER undergoes structural changes before cell division.

Structural and functional rearrangements of the ER are handled by the UPR, an evolutionarily conserved signaling network that monitors and adjusts ER function to maintain ER homeostasis.

The mammalian UPR is governed by three ER-membrane sensors that detect ER protein-folding and ER membrane perturbations, collectively known as ER stress. These ER stress sensors are the kinase/RNase IRE1, the kinase PERK, and the membrane-tethered transcription factor ATF6 (Halbleib *et al.*, 2017; Tam *et al.*, 2018; Karagöz *et al.*, 2019; Fun and Thibault, 2020). Upon ER stress sensing, IRE1 and PERK self-associate, trans-autophosphorylate, and homo-oligomerize in the plane of the ER membrane. Active IRE1 induces the transcription factor XBP1S (S for “spliced”) through nonconventional splicing of the XBP1 mRNA and degrades a subset of mRNAs in a process known as regulated IRE1-dependent decay (RIDD; Hollien and Weissman, 2006). Active PERK phosphorylates the alpha subunit of the eukaryotic translation initiation factor 2 (eIF2α), leading to a global translational shutdown and the selective translation of some mRNAs, including the stress-responsive transcription factor ATF4 (Harding *et al.*, 2000a). ER stress causes ATF6 to traffic to the Golgi apparatus, wherein it undergoes regulated proteolysis to liberate its soluble transcription factor portion from the membrane (Yoshida *et al.*, 1998). Together, IRE1, PERK, and ATF6 induce gene expression and translational programs that restore ER homeostasis (Karagöz *et al.*, 2019). XBP1 and ATF6 also regulate lipid biosynthesis and endomembrane biogenesis, thereby controlling the volumetric expansion of the ER (Sriburi *et al.*, 2004; Bommiasamy *et al.*, 2009). Because of its crucial role in ER maintenance, we reasoned the UPR could provide a long-sought housekeeping link between ER reorganization and the cell cycle that is reflective of cellular physiology.

## RESULTS

Because the UPR adjusts ER function and size, we hypothesized it could integrate ER physiology and the cell cycle. Indeed, an intact UPR is required for cytokinesis in budding yeast (Bicknell *et al.*, 2007), and, not surprisingly, activation of the UPR with ER poisons typically used in the laboratory inhibits cell cycle progression (Han *et al.*, 2013; Lee *et al.*, 2019). Nevertheless, the roles of the UPR in coordinating mammalian cell-cycle dynamics in unstressed cells remain unexamined. To address this knowledge gap, we manipulated the baseline UPR in unstressed H4 neuroglioma cells (i.e., without any acute stress induced by classical ER poisons used in the laboratory) and studied cell cycle progression. We inhibited IRE1´s RNase activity with the aldehyde inhibitor 4µ8C; ATF6 with ceapin-A7, a small molecule that prevents ATF6´s ER-to-Golgi trafficking and thus blocks its activation; and PERK with the kinase inhibitor GSK2606414 (Axten *et al.*, 2012; Cross *et al.*, 2012; Gallagher *et al.*, 2016). Because the ATF6 and IRE1-XBP1 transcriptional responses partially overlap and could be redundant (Lee *et al.*, 2003; Sriburi *et al.*, 2004; Shoulders *et al.*, 2013), we also tested a combination of 4µ8C and ceapin-A7. Because the UPR shares a sensor–PERK––with the integrated stress response (ISR), we also sought to exclude potential ISR-specific effects by treating cells with the potent ISR inhibitor ISRIB, which renders cells insensitive to the effects of eIF2α phosphorylation (Sidrauski *et al.*, 2013).

**FIGURE 1:**
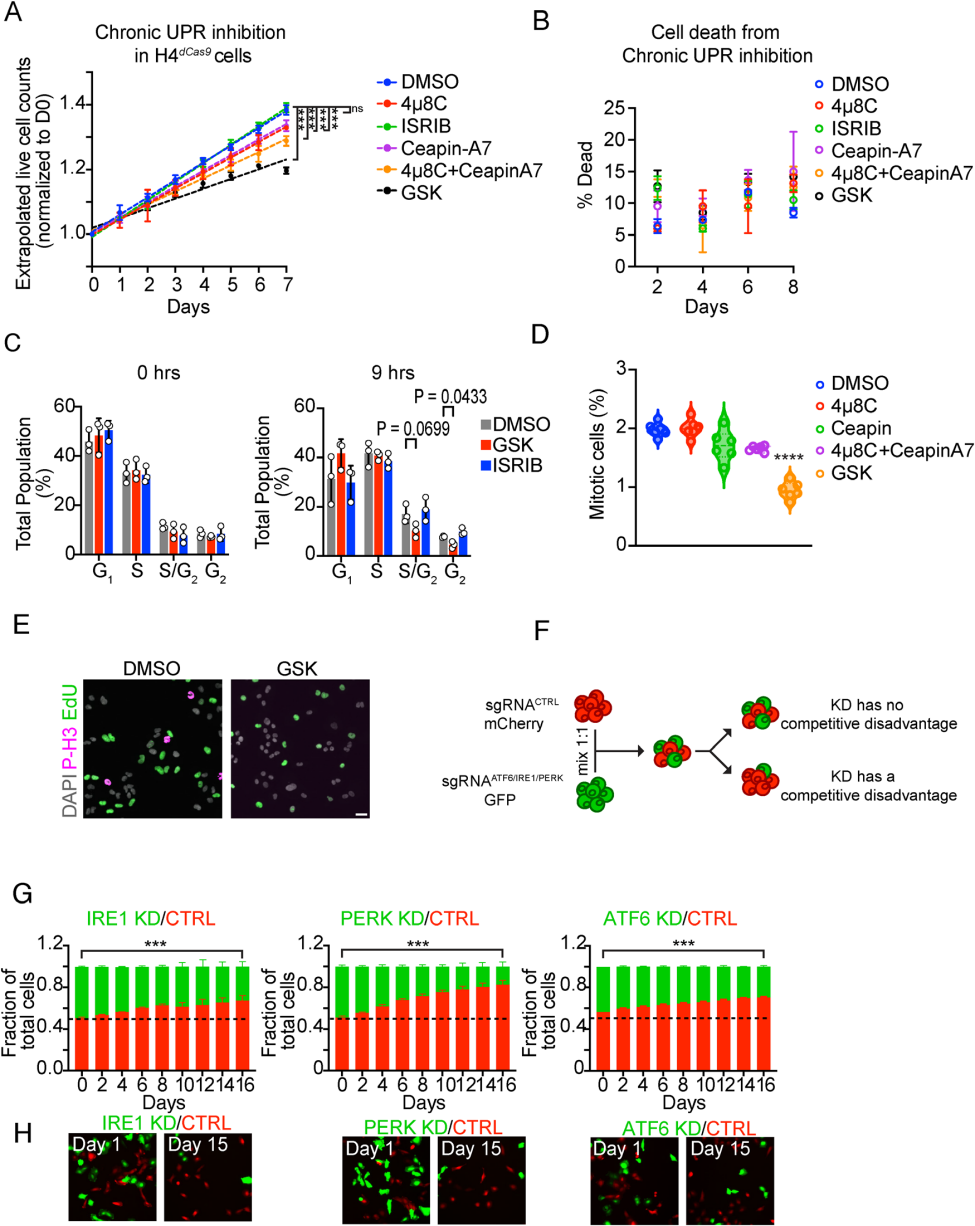
(A) Pharmacological inhibition of the UPR delays the growth of H4 CRISPRi cells (*N* = 3; Paired two-tailed *t* test, ***P < 0.001, **P < 0.01, *P < 0.05). (B) Pharmacological UPR inhibition does not induce significant cell death in H4 CRISPRi cells over one week of treatment (*N* = 3, P = n.s., Paired two-tailed *t* test; see *Materials and Methods* for details). (C) EdU/PI cell cycle profile analysis of H4 CRISPRi cells treated with GSK2606414, ISRIB, or DMSO for 4 d (0 h; see *Materials and Methods* for details) and an additional 9 h in nocodazole to arrest cells in the G_2_/M boundary (*N* = 3, two-way ANOVA). (D) Quantification and analysis of mitotic cells upon 4 d of pharmacological UPR inhibition (*N* = 5, *n* > 1377, 2-way ANOVA). (E) Representative images of the quantification in (D). Nuclei (DAPI) are shown in gray, cells stained for the mitotic marker phospho-histone-3 (P-H3) are shown in magenta, and cells stained for ethynyl-deoxyuridine (EdU), indicating DNA synthesis has occurred, are shown in green. Scale bar: 20 µm. (F) Schematic of a cell competition assay for the ratiometric measurement of the impact of the genetic depletion of each UPR sensor in cell population dynamics. (G) Flow cytometry-based quantification of the proportions of GFP+ and mCherry+ cells in the cell competition assay depicted in (F). Representative micrographs are shown below (*N* = 3; Paired two-tailed *t* test, ***P < 0.001, **P < 0.01, *P < 0.05).

Both 4µ8C and ceapin-A7 treatment reduced H4 cell proliferation over 7 d (Figure 1A), and the combination of 4µ8C and ceapin-A7 had an additive effect. To maintain drug efficacy during the time course and minimize potential effects resulting from changes in cell population density, we replenished the drugs every 24 h and, at the same time, we diluted and reseeded the cells and evaluated viability (see *Materials and Methods* for details). Surprisingly, although treatment with ISRIB was nonperturbative, GSK2606414 treatment led to a strong antiproliferative effect. Notably, none of the drugs substantially impaired cell viability in our experiments (Figure 1B), and thus, we infer that the antiproliferative effects we observed result from a delay of the cell cycle rather than induction of cell death. Moreover, a 4 d pretreatment with GSK2606414 followed by a 9-h pulse exposure to nocodazole to arrest cells in G_2_ led to a modest, yet significant, decrease in the proportion of cells in G_2_ compared with DMSO-treated control cells, as assessed by EdU pulse-labeling and EdU/PI flow cytometry profiling, while treatment with ISRIB showed no such effect (Red and blue bars compared with vehicle control grey bars at 9 h of nocodazole treatment; Figure 1C), suggesting that baseline PERK inhibition––but not ISR inhibition––delays the G_1_ to S/G_2_ transition. Analysis of mitotic cells in asynchronous populations where we inhibited the UPR sensors confirmed these observations, indicating significantly fewer cells in mitosis upon GSK2606414 treatment when compared with the control (Figure 1, D and E). Together, these results suggest that the UPR influences cell-cycle kinetics and, surprisingly, substantiate that PERK´s kinase activity, but not eIF2α phosphorylation, plays a significant role in cell cycle dynamics.

Recognizing the potential off-target effects of drugs, we set out to investigate the impact of genetic depletion of the UPR sensors on the cell cycle using a cell competition assay. We labeled H4 cells carrying the CRISPR interference (CRISPRi) machinery with GFP or mCherry. We introduced small guide RNAs (sgRNAs) targeting IRE1, PERK, or ATF6 into GFP-labeled cells, and a nontargeting (GAL4) sgRNA control into mCherry labeled cells (Figure 1F). We verified IRE1 and PERK knockdown efficiency by immunoblot, and ATF6 by qRT-PCR due to the lack of reliable antibodies (Supplemental Figure S1A). Next, we cocultured the GFP and mCherry expressing cells in a 1:1 ratio, diluted the mixed population every 48 h to maintain a constant seeding density, and analyzed the cells by flow cytometry and fluorescence microscopy at each interval. In line with our results with 4µ8C, ceapin-A7, and GSK2606414, cells depleted of IRE1, ATF6, and PERK showed a proliferative disadvantage, evidenced by a time-dependent enrichment of mCherry expressing cells (Figure 1G). Notably, the genetic depletion of the UPR sensors phenocopied the effects of the drugs, where depletion of IRE1 and ATF6 gave comparable phenotypes, while PERK depletion showed the most profound antiproliferative effect (Figure 1G), substantiating a baseline role for PERK in maintaining cell cycle dynamics.

Based on these results and considering that the UPR regulates ER expansion (Sriburi *et al.*, 2004; Bommiasamy *et al.*, 2009; Tam *et al.*, 2018), we hypothesized coordination between the observed UPR actions and ER enlargement in preparation for cell division. To measure ER expansion, we separated G_1_ or S/G_2_ H4 neuroglioma cells or KMS11 multiple myeloma CRISPRi cells by FACS based on the expression of a fluorescent reporter of cell-cycle stages known as Fast-FUCCI. This reporter encodes two fluorescent proteins, mKusibaraOrange and mAzamiGreen, fused to degrons of the cell cycle licensing factors CDT1 and geminin, respectively. The turnover of these proteins during G_1_ and G_2_ allows live cell measurements of a fluorescent label shift dependent on cell-cycle progression (Koh *et al.*, 2017). We chose H4 and KMS11 because they represent distinct physiological reliance on ER functions; KMS11 cells arise from professional secretory cells and secrete immunoglobulin light chains, while H4 cells are of neural origin with no dedicated secretory products.

**FIGURE 2:**
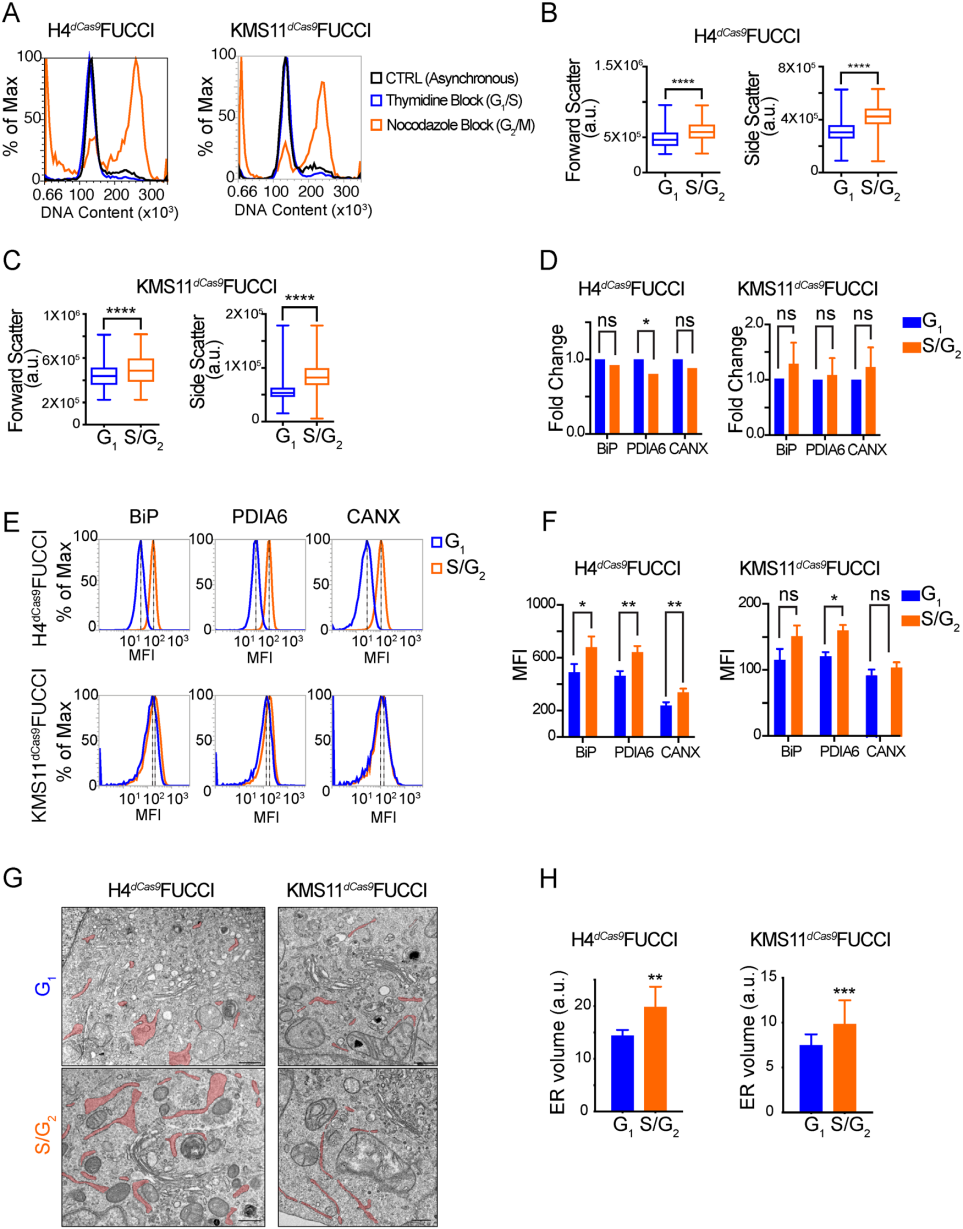
(A) Validation of the Fast-FUCCI system in CRISPRi H4 and KMS11 engineered cells by flow cytometry analysis of DNA content. The separation of G_1_/S and G_2_/M cell subpopulations was assessed upon thymidine block or nocodazole synchronization, respectively. (B and C) Side and forward scatter of asynchronous CRISPRi Fast-FUCCI cells separated by FACS according to cell cycle stage linked to the expression of the reporter. H4 N_G1_ = 37077, N_G2_ = 17202; KMS11 N_G1_ = 24919, N_G2_ = 27052; Paired two-tailed *t* test, ***P < 0.001. (D) qRT-PCR analysis of ER foldase mRNA levels in asynchronous CRISPRi Fast-FUCCI cells separated by FACS as in (B and C), (H4, left; KMS11, right), normalized to *ACTB* (*N* = 3; Paired two-tailed *t* test, *P < 0.05). (E) Flow cytometry analysis of asynchronous CRISPRi Fast-FUCCI cells separated by FACS as in (B and C) and immunostained for BiP, PDIA6, and CANX. (F) Quantification of the data in (E). MFI: mean fluorescence intensity. (*N* = 3; Paired two-tailed *t* test, **P < 0.01, *P < 0.05). (G) Representative TEM images of asynchronous G_1_ and S/G_2_ CRISPRi Fast-FUCCI cells separated by FACS as in (B and C). ER cisternae are shaded in pink. (H) Quantification of ER volume from TEM micrographs (*N* > 12 cells; Paired two-tailed *t* test, **P < 0.01, ***P < 0.001).

FACS analysis of synchronized Fast-FUCCI/CRISPRi H4 and KMS11 cell lines confirmed the validity of our reporter-based approach, as we recovered cells in distinct stages of the cell cycle (Figure 2A). Next, we separated asynchronous Fast-FUCCI/CRISPRi H4 and KMS11 cells based on G_1_ (mKusibaraOrange+) and S/G_2_ (mAzamiGreen+) reporter expression and analyzed their cell size and granularity by flow cytometry. These analyses revealed that S/G_2_ cells were larger and more granular, as indicated by increased forward and side scatter, suggesting a gain in biomass and organellar content following genome duplication (Figure 2, B and C). We next analyzed the levels of the mRNAs encoding select ER chaperones and foldases (BiP, PDIA6, and calnexin), by qRT-PCR, in FACS-separated Fast-FUCCI/CRISPRi H4 and KMS11 cells. We found no significant enrichment of these mRNAs in S/G_2_ cells compared with G_1_ cells (Figure 2D), while analysis of the corresponding proteins by immunostaining showed marked (H4) or modest (KMS11) increases in ER protein content in S/G_2_ compared with G_1_ (Figure 2, E and F). The discrepancy between mRNA and protein levels could stem from transient mRNA upregulation preceding G_2_, cell-cycle associated translational control, or differences in protein turnover. The differences in protein expression between cell lines may be linked to their intrinsic physiology, and we speculate that the myeloma cells may not need to enlarge an already expansive secretory apparatus before cell division.

Last, to investigate whether the increases in the protein levels of ER chaperones and foldases are concurrent with a volumetric ER expansion, we analyzed the ultrastructure of ER cisternae in G_1_ and S/G_2_ cells by transmission electron microscopy (TEM). To this end, we separated asynchronous H4 and KMS11 Fast-FUCCI/CRISPRi cells into G_1_ and S/G_2_ subpopulations by FACS and quantified ER cisternal volume in TEM micrographs. These analyses revealed a volumetric ER increase in S/G_2_ cells compared with those in G_1_, indicating that the ER has undergone physical expansion by the time the cells reach G_2_ (Figure 2, G and H). Consistent with our analyses of ER protein content, H4 cells showed a larger ER volumetric increase compared with KMS11 cells, supporting our interpretation of intrinsic differences in secretory capacity. These results demonstrate pervasive structural and biochemical remodeling of the ER accompanying genome duplication as cells progress from G_1_ to G_2_ and correlate with our observations of UPR involvement in the mammalian cell cycle.

## DISCUSSION

The coordination of mechanisms maintaining organelle integrity and the cell cycle is fundamental to preserving the fidelity of organelle inheritance during cell division. Our results show that blocking the UPR in unstressed cells delays cell cycle progression and that the ER undergoes dynamic changes as cells progress from G_1_ to G_2_. Our combined observations suggest the UPR likely coordinates dynamic ER changes in preparation for cell division. Our data align with previous findings showing that the UPR is required for cytokinesis in budding yeast and that increased fatty acid biosynthesis occurs during the S phase (Bicknell *et al.*, 2007; Bommiasamy *et al.*, 2009; Tam *et al.*, 2018; Merta *et al.*, 2021). From these published results and ours, it follows that the ability to expand the ER and its biosynthetic capacity is an inherent aspect of the mammalian cell cycle.

Considering that the UPR is the master surveillance system that adjusts the ER´s physiological capacity, it is likely that a housekeeping function of the UPR may be required in cycling cells for making structural and functional ER adjustments concordant with cell division. Because all three branches of the UPR respond to lipid bilayer stress and the IRE1 and ATF6 branches regulate endomembrane biosynthesis, the UPR may be particularly important for the controlled expansion of the ER we observed (Sriburi *et al.*, 2004; Bommiasamy *et al.*, 2009). In line with this notion, pharmacological and genetic inhibition of IRE1 and ATF6 delayed cell cycle progression, and their combined loss-of-function led to a more profound defect, indicating a functional redundancy that may be linked to ER expansion (Figure 1A). Surprisingly, the inhibition of IRE1 and ATF6, alone or in combination, did not amount to the extent of the effect we observed upon PERK inhibition, which suggests a housekeeping role for PERK in adjusting cell cycle timing that is inherently distinct from the potential cell cycle roles of IRE1 and ATF6.

The striking discrepancy between our results with ISRIB and PERK inhibition suggests that the baseline phosphorylation of eIF2α observed in cells not challenged by acute ER stress does not impact cell cycle dynamics to the same extent as UPR inhibition. The low levels of phosphorylated eIF2α observed in unstressed cells are likely insufficient to trigger the ISR and suppress protein synthesis, which would otherwise lead to a full-blown ISR and subsequent cell cycle arrest, arising, among other factors from suppression of the synthesis of cyclin D1, as has been previously described (Brewer and Diehl, 2000; Lee *et al.*, 2019). Moreover, ISRIB treatment boosts protein synthesis (Sidrauski *et al.*, 2013), which could facilitate the oscillatory accumulation of cyclins throughout the cell cycle, promoting its timely progression and uncoupling any potential effect of eIF2α phosphorylation levels on the periodicity of cyclin synthesis. Instead, our results suggest a specific role of PERK, upstream of eIF2α phosphorylation, in facilitating cell-cycle progression, which challenges the previous paradigm that PERK activity inhibits the mammalian cell cycle (Brewer and Diehl, 2000; Lee *et al.*, 2019). Nevertheless, this model does not need to be reevaluated, as PERK may have different activities that satisfy suppressive or permissive roles in the cell cycle, as discussed below.

Besides itself and eIF2α, PERK has been shown to phosphorylate other substrates, such as the transcription factor Nrf2 (Cullinan *et al.*, 2003), which has been proposed to be a positive regulator of the cell cycle (Lastra *et al.*, 2022). Furthermore, recent evidence indicates that PERK may play a role in phosphorylating the autophagy proteins ULK and P62, and diacylglycerol to produce phosphatidic acid, which positions PERK as a regulator of mTOR/Akt signaling–-a key cell-cycle controller (Fingar *et al.*, 2004; Bobrovnikova-Marjon *et al.*, 2012; Lee *et al.*, 2022). These observations substantiate that PERK has substrates different from eIF2α that could influence cell cycle timing and are consistent with our interpretation that PERK´s activities independent of eIF2α phosphorylation may influence cell-cycle kinetics. Our results pose an apparent dichotomy with the results of Alan Diehl, which suggest that PERK is as a negative regulator of cell cycle progression (Brewer and Diehl, 2000; Lee *et al.*, 2019). However, our results are not in conflict with this model. High PERK activity––and elevated phospho-eIF2α––elicited by PERK overexpression or treatment of cells with high doses of classical ER poisons, such as thapsigargin and tunicamycin, arrests the cell cycle in G_1_ by blocking cyclin D translation, a downstream effect of augmenting the levels of phosphorylated eIF2α and suppressing global protein synthesis (Brewer and Diehl, 2000; Harding *et al.*, 2000b; Han *et al.*, 2013; Lee *et al.*, 2019). These studies highlight a potential cytoprotective role of the UPR and the ISR in responding to acute ER stress (i.e., delaying the cell cycle until the adverse conditions are mitigated). Our data, by comparison, suggest a complementary housekeeping role of PERK, outside of its canonical role in the ISR and eIF2α phosphorylation, that fine-tunes cell cycle dynamics in the absence of acute ER stress. Collectively, our results and those of the Diehl lab highlight a fundamental aspect of the UPR in allowing cell decision-making concordant with physiological challenges: divide when conditions are favorable or the stress levels are tolerable, or halt cell division to refunnel resources and alleviate the stress.

Taken together, our data indicate that all three UPR branches have housekeeping functions in regulating cell-cycle dynamics. Whether canonical UPR inputs (i.e., ER protein-folding perturbations and lipid bilayer stress) or yet-to-be-discovered UPR-activating mechanisms regulate cell cycle dynamics is an outstanding question that warrants further investigation. Regardless of the activation mechanism, the UPR emerges as a link between ER expansion and cell cycle dynamics that may constitute a critical homeostatic checkpoint for maintaining the health of mitotic cells.

## MATERIALS AND METHODS

Request a protocol through *Bio-protocol*.

### Cell culture and drug treatments

H4 and KMS11 cells carrying the CRISPRi machinery (dCas9-KRAB; gift from Martin Kampmann, UCSF), and 293METR cells (gift from Brian Rabinovich, formerly at MD Anderson Cancer Center) were kept in DMEM (H4 and 293METR) or RPMI1640 (KMS11) supplemented with 110 mg/l sodium pyruvate, 10% fetal bovine serum (FBS), 0.1 U/ml penicillin/streptomycin, and 2 mM L-glutamine, at 37°C and 5% CO_2_ in a humidified incubator. UPR inhibitors (5 μM ceapin-A7 [Sigma], 500 nM ISRIB [MedChem Express], 10 μM 4μ8C [MedChemExpress], and 1 µM GSK2605414 [Adipogen Corp] were used for 7 d in H4 cells at a seeding density of 2.5E5 cells/well in a six-well plate. To maintain drug efficacy in experiments in which we exposed H4 cells for longer than a day, all drugs were refreshed every 24 h, an interval at which cells were diluted back to the original seeding density after cell viability measurements were taken in an automated cell counter (Countess II FL, Thermo Fisher Scientific). For experiments with KMS11 cells, the seeding density was 5E5 cells/ml (KMS11), and the subculturing and cell viability measurement routine was every 48 h.

### Plasmids, lentivirus, and retrovirus production

pBOB-EF1-FAST-FUCCI-puro (gift from Kevin Brindle and Duncan Jodrell; Addgene plasmid # 86849), and pLG15 (gift of Martin Kampman, [20]) were used to express the FUCCI reporter and sgRNAs targeting IRE1, PERK, or ATF6, respectively. The sgRNA sequences were selected from the hCRISPRi-v2 library (Jonathan Weissman lab, UCSF) and cloned as previously described (Gilbert *et al.*, 2014). Retroviral constructs encoding EGFP and mCherry were built by cloning the coding sequences of the fluorescent proteins into the pLNCX2 retroviral vector (Clontech), using standard cloning methods. Lentivirus and retrovirus production was carried out as described previously (Acosta-Alvear *et al.*, 2018).

### Flow cytometry

Fast-FUCCI cell subpopulations were selected by FACS. All FACS experiments were conducted in a Sony SH800S instrument. For flow cytometry analyses, cells were fixed in ethanol or 4% paraformaldehyde at 4°C for 15 min and stained with 50 μg/ml propidium iodide for 15 min, or with antibodies. For immunostaining, the fixed cells were resuspended in buffer FZ (50 mM NH_4_Cl, 0.5% bovine serum albumin (BSA), 0.05% saponin, 0.02% NaN_3_ in phosphate-buffered saline), and incubated at room temperature for 1 h. Primary (anti-BiP CST#3177, anti-Calnexin Abcam#ab22595, anti-PDI #3501) and fluorophore-conjugated secondary antibodies were diluted 1:500 in buffer FZ and incubated overnight at 4°C (primary) or 1 h at room temperature (secondary) with gentle agitation. All analyses were conducted on an Attune NxT Flow Cytometry (Thermo Fisher Scientific).

### Cell Synchronization

Cells were synchronized in G_1_/S using a thymidine double-block or G_2_/M by treating them with nocodazole after a first thymidine pulse, as previously described (Chen and Deng, 2018).

### Analysis of mitotic cells

H4 CRISPRi cells were pretreated for 4 d with UPR inhibitors and drug replenished after 48 h, labeled with 10 µM EdU for 30 min, fixed in 4% paraformaldehyde, permeabilized with 0.25% Triton for 10 min before click-it detection, blocked in 3% BSA, and subjected to immunostaining with antiphospho-H3 antibody (1:1000), antirabbit-Alexa594 secondary antibody (1:1000), and DAPI. Imaging was performed using a Nikon Ti-2 Eclipse inverted spinning disk confocal microscope and the micrographs were analyzed using the imaging analysis software CellProfiler (Stirling *et al.*, 2021).

### RNA extraction, cDNA synthesis, and qRT-PCR

RNA was extracted using QIAGEN´s RNeasy kit according to the manufacturer´s instructions. One microgram of DNAseI-treated RNA was reverse transcribed with the iScript cDNA synthesis system (BioRad) according to the manufacturer´s protocol. The cDNA was diluted 10-fold in nuclease-free water and 2% was used as input for SYBR green gene-specific quantitative PCR (qRT-PCR). qRT-PCRs were carried out in a CFX96 Touch qPCR instrument (BioRad), and changes in gene expression were determined using the ΔΔCq method.

### Transmission Electron Microscopy (TEM)

Cells were fixed with 1% glutaraldehyde in 0.2 M HEPES, pH-7.3 for 30 min at room temperature and postfixed in a mixture of osmium tetroxide and potassium ferrocyanide, dehydrated in ethanol and acetone, and embedded in epoxy resin as described previously (Polishchuk and Polishchuk, 2019). Thin 60-nm sections were cut using a Leica EM UC7 ultramicrotome, and EM images were acquired using a FEI Tecnai-12 electron microscope (FEI, Eindhoven, Netherlands) equipped with a VELETTA CCD digital camera (Soft Imaging Systems GmbH, Munster, Germany). ER volume quantification was performed using iTEM software (Olympus SYS, Germany) on EM images acquired at the same magnification as follows: a 700-nm morphometric grid was applied to each image, and the number of grid nodes overlapping with ER membranes and the entire cell cytoplasm was counted. ER density was calculated by dividing the number of ER-associated nodes by the number of cytoplasm nodes. The overall ER volume was calculated for each cell as mean ER density multiplied by cytoplasm volume and expressed in arbitrary units (AU).

### Cell competition assay

GFP-labeled CRISPRi H4 cells depleted of individual UPR sensors and mCherry-labeled H4 CRIPSRi cells carrying a nontargeting sgRNA control were mixed in a 1:1 ratio and diluted every 48 h to the original plating density. Samples were collected at each subculture interval for flow cytometry and microscopy analyses. Flow analyses were performed on an Attune NxT flow cytometer (Thermo Fisher Scientific) and microscopy was performed on an EchoRevolve inverted fluorescence microscope.

### EdU/PI Cell Cycle Profiling

H4 CRISPRi cells were treated with DMSO, 500 nM ISRIB, or 1 µM GSK2606414 for 4 d and drugs replenished after 48 h. Cells were labeled for 30 min with 10 µM EdU and released into 1 µM nocodazole. Cell cycle profiles were analyzed immediately after labeling or after 9 h of nocodazole treatment using PI staining as described above, and flow cytometry metrics were collected in an Attune NxT flow cytometer (Thermo Fisher Scientific).

### Immunoblotting

Lysates were collected in Laemmli sample buffer, sonicated, reduced with β-mercaptoethanol, and boiled for 5 min before loading onto BioRad TGX Stain Free 4–20% SDS–PAGE gels and transferred onto 0.45 µm PVDF membranes. Membranes were blocked for 1 h at room temperature with 3% BSA in 1X TRIS buffer saline Tween20 (TBST), then incubated at 4°C overnight in primary antibody solutions (anti-IRE1 CST#3294, anti-PERK CST#3192 diluted 1:1000 in 3% BSA, 1X TBST), washed, and incubated for 1 h in HRP-conjugated secondary antibody solution at room temperature (diluted 1 to 5000 antirabbit in 3% BSA, 1X TBST). Immunoreactive bands were detected using SuperSignal West Femto maximum sensitivity substrate (Thermo Fisher Scientific) and a ChemiDoc MP Imaging System (BioRad).

## Supplementary Material


