# Supplementary material for: Baseline unfolded protein response signaling adjusts the timing of the mammalian cell cycle
Source: Mol Biol Cell. 2024 May 20;35(6):br12. doi: 10.1091/mbc.E23-11-0419 (PMC11238080; doi:10.1091/mbc.E23-11-0419)
Supplement: Supplementary file 1 [file mbc-35-br12-s001.pdf]

Supplemental Materials

Molecular Biology of the Cell

Chowdhury *et al*.

Supplemenary Figure 1. (A) Western blot (left) and qRT-PCR (right) analyses of IRE1, PERK, and ATF6 levels upon CRISPRi knockdown in H4 cells. Densitometry quantification is shown below blots.

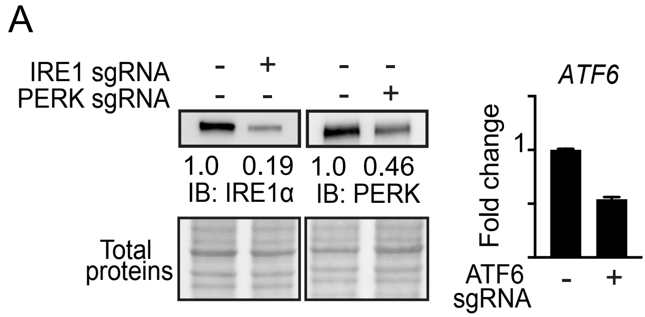
A
IRE1 sgRNA - + - -
PERK sgRNA - - - +
1.0 0.19 1.0 0.46
IB: IRE1α IB: PERK
Total proteins
ATF6
Fold change
1
ATF6 sgRNA - +

Figure S1
