# Supplementary material for: Baseline unfolded protein response signaling adjusts the timing of the mammalian cell cycle
Source: Mol Biol Cell. 2024 May 20;35(6):br12. doi: 10.1091/mbc.E23-11-0419 (PMC11238080; doi:10.1091/mbc.E23-11-0419)
Supplement: Supplementary file 2 [file reviewer_reportE23-11-0419.pdf]

Baseline Unfolded Protein Response Signaling Adjusts the Timing of the Mammalian Cell Cycle

Soham Chowdhury, Sabrina Solley, Elena Polishchuk, Julien Bacal, Julia Conrad, Brooke Gardner, Diego Acosta Alvear, and Francesca Zappa

Corresponding author(s): Francesca Zappa, Altos Labs Inc

Review Timeline:

Submission Date:	2023-11-06
Editorial Decision:	2023-11-28
Revision Received:	2024-02-16
Editorial Decision:	2024-03-27
Revision Received:	2024-04-10
Editorial Decision:	2024-04-11
Revision Received:	2024-04-15
Accepted:	2024-04-16

Editor-in-Chief: Matthew Welch

Transaction Report:

(Note: With the exception of the correction of typographical or spelling errors that could be a source of ambiguity, letters and reports are not edited. The original formatting of letters and referee reports may not be reflected in this compilation.)

RE: Manuscript #E23-11-0419
TITLE: "The Unfolded Protein Response Regulates the Mammalian Cell Cycle"

Dear Dr. Zappa,

Your manuscript, entitled "The Unfolded Protein Response Regulates the Mammalian Cell Cycle," has been evaluated by two referees, whose detailed comments are included for your reference. Both referees acknowledged the interest in your findings yet expressed concerns regarding the study's limited scope. Key issues highlighted include the differing outcomes when inhibiting PERK and its downstream target eIF2α, and the necessity to assess the effects of UPR inhibitors during mitosis. Additionally, there is concern about discrepancies between your findings and previous reports, which have not been addressed in your manuscript. Given the need for significant additional experiments to substantiate your conclusions, we regret to inform you that we cannot accept your paper for publication in its current form. However, we would be willing to consider your manuscript for a new submission after substantial revision and new data addressing the major concerns of the referees.

Thank you for the opportunity to examine this work. We hope that as your studies progress you will consider submitting future manuscripts to Molecular Biology of the Cell (MBoC).

If you have any questions regarding the decision, please contact the MBoC Editorial Office (mboc@ascb.org).

Sincerely,
Guillaume Thibault
Monitoring Editor
Molecular Biology of the Cell

Reviewer #1 (Remarks to the Author):

This Brief Report "The Unfolded Protein Response Regulates the Mammalian Cell Cycle" seeks to investigate effects of inhibition of UPR sensor proteins IRE1, ATF6 and PERK on the cell cycle. Among the novel findings: The authors found that the cell cycle is slowed down, with PERK kinase activity found to have the most significant effect on cell proliferation from the inhibition experiments (Fig. 1). To assess potential coordination between the observed UPR action and ER enlargement, in preparation for mitotic entry, ER expansion was analysed in facs-sorted cells from synchronised experiments, with discernible observation of ER enlargement prior to mitosis (Fig. 2). This represents novel finding for EM and potential implications in homeostatic control for mitosis. Appropriate cells and synchronisation experiments, and inhibitors have been used.
Comments:
(1) Since ER may potentially affect mitosis, duration of mitosis should be assessed upon UPR sensor inhibition. i.e. time-lapse at nuclear breakdown to nuclear reformation, to assess possible differences compared to control.
(2) Current title is too broad and not supported by findings. The title should be changed and point to regulation of a specific aspect of the cell cycle.

Reviewer #2 (Remarks to the Author):

See attached.

Dear Dr. Thibault,

We are grateful for handling and thoroughly evaluating our manuscript, "The Unfolded Protein Response Regulates the Mammalian Cell Cycle," with reference number #E23-11-0419. We are thankful for the insightful feedback. We have carefully considered and addressed the comments and made corresponding revisions to our manuscript for your consideration.

Reviewer #1:

(1) *Since ER may potentially affect mitosis, duration of mitosis should be assessed upon UPR sensor inhibition i.e. time-lapse at nuclear breakdown to nuclear reformation, to assess possible differences compared to control.*

We completely agree. To provide a complete picture of the effects of UPR modulation in unstressed cycling cells, we measured mitotic events following the pharmacological inhibition of the individual UPR ER stress sensors, IRE1, PERK, and ATF6. Consistent with our findings that PERK inhibition negatively impacts cell cycle progression, we observed that treatment with the PERK inhibitor GSK2606414 significantly decreased mitotic entry in cells not treated with ER stress-inducing drugs (new panel Figure 1D, E). These findings support our interpretation that baseline PERK activity is critical for adjusting cell cycle timing.

(2) Current title is too broad and not supported by findings. The title should be changed and point to regulation of a specific aspect of the cell cycle.

We agree with the recommendations and have revised the title to "Baseline Unfolded Protein Response Signaling Adjusts the Timing of the Mammalian Cell Cycle." We thank the reviewer for this insightful suggestion.

We appreciate the constructive comments provided by Reviewer #1.

Reviewer #2:

In 2000, Alan Diehl and his colleagues reported that the activation of PERK by ER stress results in G1 cell-cycle arrest. This arrest is, at least in part, caused by reduced levels of cyclin D1 due to a decrease in global translation mediated by PERK-induced phosphorylation of eIF2a. The authors should perform further experiments to address this discrepancy. Furthermore, the authors must cite Alan Diehl's work and provide an explanation for the differing results.

We don’t understand this comment. We cited and discussed the apparent dichotomy between Alan Diehl's and colleagues' previous results and ours in the previous version (Discussion lines 199-204 in the previous manuscript, ref #34). In response to the Reviewer’s remarks, we have

extended the discussion accordingly (Discussion, lines 218-243). To reiterate, we attribute the dichotomy to the simple fact that we inhibited endogenous PERK in unstressed cells, whereas Diehl et al. investigated cell cycle effects after induction of ER stress or upon over-expression of PERK, leading to its constitutive activation.

Consistent with this resolution of the discrepancy, we find that in H4 cell lines stably expressing a phosphomimetic eIF2α S51D, i.e., ISR forced-ON, cyclin D levels are reduced (see figure below). While resolving the discrepancy, we feel that these data are a diversion and not in line with the scope of this paper. We, therefore, prefer not to include them.

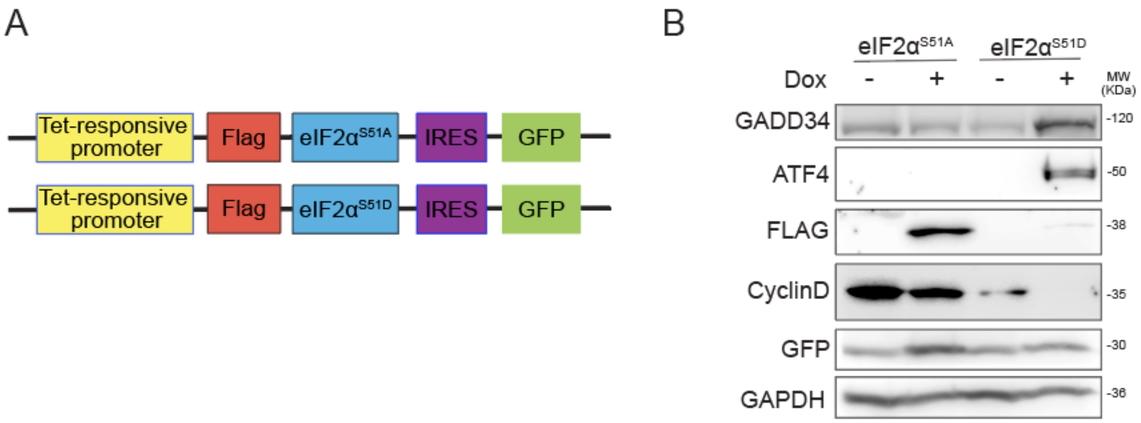

Schematic of the plasmids used in this study. B. Western blot analysis of H4 cells stably expressing eIF2α^{S51A} and eIF2α^{S51D} and treated with 300ng/µl of Doxycycline for 24hrs.

Furthermore, both the introduction and discussion in the current manuscript are too general and read more like a part of an introduction and discussion section of a review. In addition, many relevant references are missing, including those described above. Overall, significant changes are needed to be considered for publication in Molecular Biology of the Cell (MBOC).

Again, we feel we accurately cited the relevant work as explained above. In response to the Reviewer's concerns, we have added a few more references. Considering the editorial priorities of MBoC, we prefer to keep our narrative accessible, aiming to disseminate and communicate science to a broader audience.

We hope the updated version of our manuscript is appropriate for publication in MBoC.

We thank you for your efforts and for reconsidering our work.

Sincerely,

Francesca and Diego

RE: Manuscript #E23-11-0419R-A
TITLE: "Baseline Unfolded Protein Response Signaling Adjusts the Timing of the Mammalian Cell Cycle"

Dear Dr. Zappa:

Thank you for resubmitting your manuscript. We have received feedback from the reviewers on your revised submission. While Reviewer #1 is satisfied with the revisions you have made, Reviewer #2 still raises significant concerns regarding the preliminary nature of your study and the need for further clarity and detail in both the experimental descriptions and the data analysis presented.

Given the focus of this submission as a brief report, we aim to keep the requirement for new experiments to a minimum, emphasizing instead the importance of addressing the reviewers' comments through modifications in the manuscript's writing. Specifically:

Clarity and Detail: Reviewer #2 points out a need for more detailed information in the Results section, figure legends, and Materials and Methods. This includes clarifying the efficacy of UPR inhibitors over extended durations and addressing the potential variability in UPR component activities across different cell types. We ask that you provide more explicit descriptions of your experimental conditions and methodologies to enable readers to assess the validity of your results more effectively.

Data Analysis and Alignment: There appears to be a misalignment between the experimental results shown and the subsequent data analyses, particularly noted in Figures 2E and 2F. Please review these sections to ensure that the quantitation accurately reflects the presented FACS data and that the methodology for cell cycle population separation is clearly described.

Inclusion of Relevant Literature: The lack of clear mention of prior findings, specifically the work by Diehl's lab on PERK-dependent eIF2α phosphorylation, was noted. We encourage you to integrate a discussion of relevant literature more thoroughly, particularly where it directly impacts the interpretation of your findings. This will not only enhance the manuscript's contextual relevance but also strengthen the narrative of your research story.

In light of these comments, we believe that careful attention to these areas of the manuscript can address the current concerns without necessitating extensive new experiments. However, where Reviewer #2 has explicitly requested experimental data to substantiate claims (if any such specific requests were made), we kindly ask you to consider these suggestions carefully and incorporate them where feasible and deemed necessary.

Please submit your revised manuscript along with a detailed response to the reviewers' comments, outlining how each point has been addressed or explaining the rationale for any comments not addressed.

We look forward to receiving your revised manuscript.

Sincerely,
Guillaume Thibault
Monitoring Editor
Molecular Biology of the Cell

P.S. Please divide the Significance Statement into three bullet points as follows:

The endoplasmic reticulum (ER) is a single-copy organelle that cannot be generated de novo, and its functions are controlled by the Unfolded Protein Response (UPR). How the UPR impacts the cell cycle remained unclear.

By investigating this connection, the authors describe distinct roles for each UPR sensor, with PERK activity significantly affecting cell cycle beyond its typical function in the Integrated Stress Response (ISR). They also show that the ER expands before mitosis, potentially linking ER expansion to partitioning during mitosis.

These findings highlight an unexplored UPR function in cell cycle regulation, potentially serving as a critical checkpoint for mitosis.

Dear Dr. Zappa,

The review of your manuscript, referenced above, is now complete. The Monitoring Editor has decided that your manuscript

requires minor revisions before it can be published in Molecular Biology of the Cell, as described in the Monitoring Editor's decision letter above and the reviewer comments (if any) below.

A reminder: Please do not contact the Monitoring Editor directly regarding your manuscript. If you have any questions regarding the review process or the decision, please contact the MBoC Editorial Office (mboc@ascb.org).

When submitting your revision include a rebuttal letter that details, point-by-point, how the Monitoring Editor's and reviewers' comments have been addressed. (The file type for this letter must be "rebuttal letter"; do not include your response to the Monitoring Editor and reviewers in a "cover letter.") Please bear in mind that your rebuttal letter will be published with your paper if it is accepted, unless you have opted out of publishing the review history.

Authors are allowed 15 days to submit a revision. If this time period is inadequate, please contact us immediately at mboc@ascb.org.

In preparing your revised manuscript, please follow the instruction in the Information for Authors (www.molbiolcell.org/info-for-authors). In particular, to prepare for the possible acceptance of your revised manuscript, submit final, publication-quality figures with your revision as described.

To submit the rebuttal letter, revised version, and figures, please use this link (please enable cookies, or cut and paste URL):
Link Not Available

MBoC offers the option to publish your work with immediate open access. Open access can increase the discoverability and usability of your research. If you would like to publish your paper with immediate open access but did not select this option during initial submission, please contact us at mbc@ascb.org..

Authors of Articles and Brief Communications whose manuscripts have returned for minor revision ("revise only") are encouraged to create a short video abstract to accompany their article when it is published. These video abstracts, known as Science Sketches, are up to 2 minutes long and will be published on YouTube and then embedded in the article abstract. Science Sketch Editors on the MBoC Editorial Board will provide guidance as you prepare your video. Information about how to prepare and submit a video abstract is available at www.molbiolcell.org/science-sketches. Please contact mboc@ascb.org if you are interested in creating a Science Sketch.

Thank you for submitting your manuscript to MBoC. Please do not hesitate to contact this office if you have any questions.

Sincerely,

Eric Baker
Journal Production Manager
MBoC Editorial Office
mbc@ascb.org

Reviewer #1 (Remarks to the Author):

Authors have addressed my comments to my satisfaction.

Reviewer #2 (Remarks to the Author):

See Attached.

The resubmitted manuscript remains too preliminary for the caliber of work typically published in *Molecular Biology of the Cell (MBoC*). Significant changes are needed, particularly the addition of new experimental data to extend our current understanding of the roles of UPR during the cell cycle. While it serves as a starting point for further work, revisions are required to meet the journal's standards. Additionally, detailed information regarding figure panels is missing from the Results section, figure legends, and Materials and Methods, making it difficult to assess the validity of the experimental results.

We have included more detailed information in the results section and figure legends, and expanded the Materials and Methods section, adding more detailed experimental procedures to substantiate the validity of our experimental results, as described below.

For example, the data presented in Figure 1A-B indicate that previously published chemical UPR inhibitors have an impact on cell growth over a period of a week. However, it remains unclear how effective any of these UPR inhibitors are after an extended duration. Additionally, not only could the expression levels vary, but also the activities of the three UPR components, IRE1, PERK, and ATF6, could differ depending upon the specific cell type, leading to potential differences in the efficacies of each UPR inhibitor accordingly. This becomes particularly important if cells were treated for extended period. No detailed descriptions of the experiments, including how cells were maintained over seven (or longer) days in the presence of UPR inhibitors, were provided within the manuscript, making it challenging to draw general conclusions. For example, how effective were the drugs after prolonged exposure?

In the Material and Methods section in our resubmitted manuscript, we included details about cell sub-culturing routines and drug administration (lines 262-266), *"UPR inhibitors (5 μM ceapin-A7 [Sigma], 500 nM ISRIB [MedChem Express], 10 μM 4μ8C [MedChemExpress], and 1 μM GSK2605414 [Adipogen Corp] were used for 7 days at seeding densities of 2.5E5 cells/well in a 6-well plate (H4), or 5E5 cells/mL (KMS11), and the viable cell number was measured every 24 h (H4) or every 48 h (KMS11) in an automated cell counter (Countess II FL, ThermoFisher)."* We recognize this limited description may have led to confusion. Thus, we have rephrased this section as follows (lines 289-296): "UPR inhibitors (5 μM ceapin-A7 [Sigma], 500 nM ISRIB [MedChem Express], 10 μM 4μ8C [MedChemExpress], and 1 μM GSK2605414 [Adipogen Corp] were used for 7 days in H4 cells at a seeding density of 2.5E5 cells/well in a 6-well plate. To maintain drug efficacy in experiments in which we exposed H4 cells for longer than a day, all drugs were refreshed every 24 hours, an interval at which cells were diluted back to the original seeding density after cell viability measurements were taken in an automated cell counter (Countess II FL, Thermo). For experiments with KMS11 cells, the seeding density was 5E5 cells/mL (KMS11), and the subculturing and cell viability measurement routine was every 48 h." We have also specified drug replenishment in lines 323 and 359.

Regarding the expression levels of UPR components, we expect these may vary in different cell types, and for this reason, we performed most of our experiments in parallel using two cell types, KMS11 multiple myeloma (MM) cells and H4 neuroglioma cells, representing different ontogeny, physiology, and UPR reliance. MM cells are recognized to be reliant on UPR activity due to their intrinsic secretory nature (PMIDs: 16507771; 27284736; 31371506), while H4 cells were used as a more general model system which, nevertheless, have been extensively used in neuroscience research (PMIDs: 32296178; 450131). The drugs employed in this study have been extensively characterized elsewhere (PMIDs: 27435962; 27435960; 25719440; 22315414; 22827572), and the drug concentrations we used fall within standard use ranges for cells in culture to attenuate UPR activity.

We also included details in the Results section, in lines 128-131, 135-136.

It is also reasonable to consider that each inhibitor used for an extended duration may affect target proteins beyond IRE1, PERK, and ATF6, or off-target components. Once again, no follow-up experiments were included to demonstrate the validity of the experimental design. Although experiments shown in Figure 1G and 1H begin to address this issue, it is too preliminary to draw clear conclusions beyond the importance of UPR components for cell growth, which may or may not be directly related to the cell cycle.

We recognize the potential off-target effects elicited by small molecule UPR inhibitors, and, for this reason, we validated our results obtained using small molecule inhibitors with genetics-based approaches, which we stated in lines 147-153 of our resubmitted manuscript. These genetics-based data (Figures 1F-H) mirror the results we obtained with the pharmacological UPR inhibitors, which corroborates the direct involvement of the UPR sensors in modulating cell proliferation (additional interpretation of our data in the Results section in lines 158-161). As far as we know, genetic knock-downs remain one of the most stringent and rigorously validated approaches for evaluating target-specific effects (PMIDs: 33420254; 15555080; 33536628).

In addition, the data analyses do not align with the experimental results shown. For example, in Figure 2E and F, it states that quantitation of 2E is shown in 2F. However, the FACS data presented in 2E and the quantitation in 2F for BiP, PDIA6, and CANX, do not seem to align, specifically in KMS11 $_{\text{dcas9}}$FUCCI cells. Additionally, how were the cell cycle populations separated for the experiments described in each panel? Were cells released from the drug upon double thymidine or thymidine/nocodazole synchronization? Were synchronized cells used immediately after release into the cell cycle, or was there any time allotted for recovery from thymidine or nocodazole? Furthermore, for nocodazole-synchronized cells, there are instances of mixed G1/S phase cells within the G2/M phase, and the ratio of G1/S cells to G2/M cells differs between the H4 $_{\text{dcas9}}$FUCCI and KMS11 $_{\text{dcas9}}$FUCCI cell lines. This suggests that the durations of cell cycle phases often vary between different cell lines. Without considering such differences, it is difficult to assess the significance of the data presented in Figure 2E and F. Furthermore, the authors do not provide any significant interpretations or conclusions beyond simply stating the results.

The data quantification depicted in Figure 2F corresponds to the median fluorescent intensity (MFI) determined through flow cytometry analysis, illustrated in Figure 2E. All experiments presented in Figure 1 utilized cells expressing the FUCCI reporter, an extensively validated tool that allows separating cells in different stages of the cell cycle by flow cytometry (PMIDs: 27888217; 32957652). With the exception of the data shown in panel 2A, which validates the FUCCI system in our hands (Fig. 2A), no cell cycle arrest induced by nocodazole or thymidine was applied to the rest of the data presented in Figure 2, which we have clarified in lines 176-180, 196, and in the Figure legend.

Because pharmacological cell cycle arrest carries the risk of drug off-target effects that may impinge on UPR activity and confound the interpretation of our results, we relied on the FUCCI reporter, and we gated on G_1 and G_2/S populations as described in the text and figure captions in panels 2B-H. Differences in cell cycle timing are to be expected when working with different cell lines. However, we do not think such differences murk or negate our conclusions.

Finally, I would like to point out that in the previous version of manuscript, there was no clear description of PERK-dependent eIF2□ _phosphorylation-dependent translation repression, which induced the reduction of cyclin D1 and subsequently diminished the activity of the cell cycle-

dependent kinase, as previously reported by the Diehl lab. The inclusion of previously published work within the manuscript without a clear mention of these prior findings, especially since Diehl's work directly addresses the impact of one of the UPR components on the cell cycle, diminishes the importance of drawing readers' attention to relevant literature. Simply citing their paper without clarification is not sufficient.

We included and discussed Alan Diehl's published works in our resubmitted manuscript. However, we have expanded our discussion to further clarify how these important contributions relate to our work (lines 235-237, 255-270).

In the revised abstract, the authors now include a sentence stating that "this effect results from the slowdown of the G1-to-S/G2 transition, which depends on PERK's kinase activity." However, previous work goes beyond "its well characterized role in protein synthesis regulation through the phosphorylation of eIF2α*,*" as mentioned above, regarding how eIF2a-induced translation repression impacts cyclin D1 levels.

For clarity, we have rephrased the abstract (lines 39-45), which now reads "Here, we show that pharmacological and genetic inhibition of the UPR sensors IRE1, ATF6, and PERK in unstressed cells delays the cell cycle, with PERK inhibition showing the most penetrant effect, which was associated with a slowdown of the G_1-to-S/G_2 transition. Treatment with the small molecule ISRIB to bypass the effects of PERK-dependent phosphorylation of the translation initiation factor eIF2α had no such effect, suggesting that cell cycle timing depends on PERK's kinase activity but is independent of eIF2α phosphorylation."

Finally, it is expected that PERK activation might be quickly turned off or neutralized by eIF2□ _phosphatase induction, as reported by several groups. Over a long time course (even 9 hours later), it might be challenging to observe, but this does not mean that it is not involved.

We agree, and therefore, we subjected cells to prolonged exposure to ISRIB (7 days), with daily drug replenishment. ISRIB is a potent ISR inhibitor (EC50 of 5nM) that renders cells insensitive to the effects of eIF2α phosphorylation, including GADD34 feedback control (PMID: 31402619). While our data—and interpretation of our results—do not rule out a potential effect of ISR feedback control in our experiments, any transient phosphorylation of eIF2α would be counteracted by ISRIB, and therefore, a major contribution of ISR feedback control remains unlikely.

Overall, experiments in Figure 1 and 2 may lay the foundation for developing a story with significant and concrete conclusions. However, the current resubmitted manuscript, despite limited editorial changes, fails to provide a sufficient advancement in our understanding of the UPR's role in the cell cycle and does not meet the caliber of papers typically published in *MBoC*.

We hope that these revisions to our manuscript are sufficient to clarify the concerns that were noted. We thank the reviewer for their time.

RE: Manuscript #E23-11-0419RR
TITLE: "Baseline Unfolded Protein Response Signaling Adjusts the Timing of the Mammalian Cell Cycle"

Dear Dr. Zappa:

Thank you for revising your manuscript, entitled "Baseline Unfolded Protein Response Signaling Adjusts the Timing of the Mammalian Cell Cycle", in response to the reviewers' comments. I believe that the modifications have improved the clarity of your manuscript. We will be pleased to publish your manuscript in MBoC once the significant statement has been modified according to MBoC guidelines.

The Significance Statement should include 3 bullet points (maximum of 100 words total) describing the main conclusions of the article and its significance for the field. These statements should be more concise and broadly accessible than the abstract, written for a general scientific audience to provide some context for the potential impact of the work. Significance Statements should be structured as following:

What is the background context? What gap in knowledge does this study address?
What are the key findings? What is unique or new about the approach?
Why is this paper significant? How might it influence future research?

More information at https://www.molbiolcell.org/curation-tools

Sincerely,
Guillaume Thibault
Monitoring Editor
Molecular Biology of the Cell

Dear Dr. Zappa,

The review of your manuscript, referenced above, is now complete. The Monitoring Editor has decided that your manuscript requires minor revisions before it can be published in Molecular Biology of the Cell, as described in the Monitoring Editor's decision letter above and the reviewer comments (if any) below.

A reminder: Please do not contact the Monitoring Editor directly regarding your manuscript. If you have any questions regarding the review process or the decision, please contact the MBoC Editorial Office (mboc@ascb.org).

When submitting your revision include a rebuttal letter that details, point-by-point, how the Monitoring Editor's and reviewers' comments have been addressed. (The file type for this letter must be "rebuttal letter"; do not include your response to the Monitoring Editor and reviewers in a "cover letter.") Please bear in mind that your rebuttal letter will be published with your paper if it is accepted, unless you have opted out of publishing the review history.

Authors are allowed 15 days to submit a revision. If this time period is inadequate, please contact us immediately at mboc@ascb.org.

In preparing your revised manuscript, please follow the instruction in the Information for Authors (www.molbiolcell.org/info-for-authors). In particular, to prepare for the possible acceptance of your revised manuscript, submit final, publication-quality figures with your revision as described.

To submit the rebuttal letter, revised version, and figures, please use this link (please enable cookies, or cut and paste URL): Link Not Available

MBoC offers the option to publish your work with immediate open access. Open access can increase the discoverability and usability of your research. If you would like to publish your paper with immediate open access but did not select this option during initial submission, please contact us at mbc@ascb.org..

Authors of Articles and Brief Communications whose manuscripts have returned for minor revision ("revise only") are encouraged to create a short video abstract to accompany their article when it is published. These video abstracts, known as Science Sketches, are up to 2 minutes long and will be published on YouTube and then embedded in the article abstract. Science Sketch Editors on the MBoC Editorial Board will provide guidance as you prepare your video. Information about how to

prepare and submit a video abstract is available at www.molbiolcell.org/science-sketches. Please contact mboc@ascb.org if you are interested in creating a Science Sketch.

Thank you for submitting your manuscript to MBoC. Please do not hesitate to contact this office if you have any questions.

Sincerely,

Eric Baker
Journal Production Manager
MBoC Editorial Office
mbc@ascb.org

The resubmitted manuscript remains too preliminary for the caliber of work typically published in *Molecular Biology of the Cell (MBoC*). Significant changes are needed, particularly the addition of new experimental data to extend our current understanding of the roles of UPR during the cell cycle. While it serves as a starting point for further work, revisions are required to meet the journal's standards. Additionally, detailed information regarding figure panels is missing from the Results section, figure legends, and Materials and Methods, making it difficult to assess the validity of the experimental results.

We have included more detailed information in the results section and figure legends, and expanded the Materials and Methods section, adding more detailed experimental procedures to substantiate the validity of our experimental results, as described below.

For example, the data presented in Figure 1A-B indicate that previously published chemical UPR inhibitors have an impact on cell growth over a period of a week. However, it remains unclear how effective any of these UPR inhibitors are after an extended duration. Additionally, not only could the expression levels vary, but also the activities of the three UPR components, IRE1, PERK, and ATF6, could differ depending upon the specific cell type, leading to potential differences in the efficacies of each UPR inhibitor accordingly. This becomes particularly important if cells were treated for extended period. No detailed descriptions of the experiments, including how cells were maintained over seven (or longer) days in the presence of UPR inhibitors, were provided within the manuscript, making it challenging to draw general conclusions. For example, how effective were the drugs after prolonged exposure?

In the Material and Methods section in our resubmitted manuscript, we included details about cell sub-culturing routines and drug administration (lines 262-266), *"UPR inhibitors (5 μM ceapin-A7 [Sigma], 500 nM ISRIB [MedChem Express], 10 μM 4μ8C [MedChemExpress], and 1 μM GSK2605414 [Adipogen Corp] were used for 7 days at seeding densities of 2.5E5 cells/well in a 6-well plate (H4), or 5E5 cells/mL (KMS11), and the viable cell number was measured every 24 h (H4) or every 48 h (KMS11) in an automated cell counter (Countess II FL, ThermoFisher).*" We recognize this limited description may have led to confusion. Thus, we have rephrased this section as follows (lines 289-296): "UPR inhibitors (5 μM ceapin-A7 [Sigma], 500 nM ISRIB [MedChem Express], 10 μM 4μ8C [MedChemExpress], and 1 μM GSK2605414 [Adipogen Corp] were used for 7 days in H4 cells at a seeding density of 2.5E5 cells/well in a 6-well plate. To maintain drug efficacy in experiments in which we exposed H4 cells for longer than a day, all drugs were refreshed every 24 hours, an interval at which cells were diluted back to the original seeding density after cell viability measurements were taken in an automated cell counter (Countess II FL, Thermo). For experiments with KMS11 cells, the seeding density was 5E5 cells/mL (KMS11), and the subculturing and cell viability measurement routine was every 48 h." We have also specified drug replenishment in lines 323 and 359.

Regarding the expression levels of UPR components, we expect these may vary in different cell types, and for this reason, we performed most of our experiments in parallel using two cell types, KMS11 multiple myeloma (MM) cells and H4 neuroglioma cells, representing different ontogeny, physiology, and UPR reliance. MM cells are recognized to be reliant on UPR activity due to their intrinsic secretory nature (PMIDs: 16507771; 27284736; 31371506), while H4 cells were used as a more general model system which, nevertheless, have been extensively used in neuroscience research (PMIDs: 32296178; 450131). The drugs employed in this study have been extensively characterized elsewhere (PMIDs: 27435962; 27435960; 25719440; 22315414; 22827572), and the drug concentrations we used fall within standard use ranges for cells in culture to attenuate UPR activity.

We also included details in the Results section, in lines 128-131, 135-136.

It is also reasonable to consider that each inhibitor used for an extended duration may affect target proteins beyond IRE1, PERK, and ATF6, or off-target components. Once again, no follow-up experiments were included to demonstrate the validity of the experimental design. Although experiments shown in Figure 1G and 1H begin to address this issue, it is too preliminary to draw clear conclusions beyond the importance of UPR components for cell growth, which may or may not be directly related to the cell cycle.

We recognize the potential off-target effects elicited by small molecule UPR inhibitors, and, for this reason, we validated our results obtained using small molecule inhibitors with genetics-based approaches, which we stated in lines 147-153 of our resubmitted manuscript. These genetics-based data (Figures 1F-H) mirror the results we obtained with the pharmacological UPR inhibitors, which corroborates the direct involvement of the UPR sensors in modulating cell proliferation (additional interpretation of our data in the Results section in lines 158-161). As far as we know, genetic knock-downs remain one of the most stringent and rigorously validated approaches for evaluating target-specific effects (PMIDs: 33420254; 15555080; 33536628).

In addition, the data analyses do not align with the experimental results shown. For example, in Figure 2E and F, it states that quantitation of 2E is shown in 2F. However, the FACS data presented in 2E and the quantitation in 2F for BiP, PDIA6, and CANX, do not seem to align, specifically in KMS11 $_{dcas9}$FUCCI cells. Additionally, how were the cell cycle populations separated for the experiments described in each panel? Were cells released from the drug upon double thymidine or thymidine/nocodazole synchronization? Were synchronized cells used immediately after release into the cell cycle, or was there any time allotted for recovery from thymidine or nocodazole? Furthermore, for nocodazole-synchronized cells, there are instances of mixed G1/S phase cells within the G2/M phase, and the ratio of G1/S cells to G2/M cells differs between the H4 $_{dcas9}$FUCCI and KMS11 $_{dcas9}$FUCCI cell lines. This suggests that the durations of cell cycle phases often vary between different cell lines. Without considering such differences, it is difficult to assess the significance of the data presented in Figure 2E and F. Furthermore, the authors do not provide any significant interpretations or conclusions beyond simply stating the results.

The data quantification depicted in Figure 2F corresponds to the median fluorescent intensity (MFI) determined through flow cytometry analysis, illustrated in Figure 2E. All experiments presented in Figure 1 utilized cells expressing the FUCCI reporter, an extensively validated tool that allows separating cells in different stages of the cell cycle by flow cytometry (PMIDs: 27888217; 32957652). With the exception of the data shown in panel 2A, which validates the FUCCI system in our hands (Fig. 2A), no cell cycle arrest induced by nocodazole or thymidine was applied to the rest of the data presented in Figure 2, which we have clarified in lines 176-180, 196, and in the Figure legend.

Because pharmacological cell cycle arrest carries the risk of drug off-target effects that may impinge on UPR activity and confound the interpretation of our results, we relied on the FUCCI reporter, and we gated on G_1 and G_2/S populations as described in the text and figure captions in panels 2B-H. Differences in cell cycle timing are to be expected when working with different cell lines. However, we do not think such differences murk or negate our conclusions.

Finally, I would like to point out that in the previous version of manuscript, there was no clear description of PERK-dependent eIF2□ _phosphorylation-dependent translation repression, which induced the reduction of cyclin D1 and subsequently diminished the activity of the cell cycle-

dependent kinase, as previously reported by the Diehl lab. The inclusion of previously published work within the manuscript without a clear mention of these prior findings, especially since Diehl's work directly addresses the impact of one of the UPR components on the cell cycle, diminishes the importance of drawing readers' attention to relevant literature. Simply citing their paper without clarification is not sufficient.

We included and discussed Alan Diehl's published works in our resubmitted manuscript. However, we have expanded our discussion to further clarify how these important contributions relate to our work (lines 235-237, 255-270).

In the revised abstract, the authors now include a sentence stating that "this effect results from the slowdown of the G1-to-S/G2 transition, which depends on PERK's kinase activity." However, previous work goes beyond "its well characterized role in protein synthesis regulation through the phosphorylation of eIF2α," as mentioned above, regarding how eIF2a-induced translation repression impacts cyclin D1 levels.

For clarity, we have rephrased the abstract (lines 39-45), which now reads "Here, we show that pharmacological and genetic inhibition of the UPR sensors IRE1, ATF6, and PERK in unstressed cells delays the cell cycle, with PERK inhibition showing the most penetrant effect, which was associated with a slowdown of the G_1-to-S/G_2 transition. Treatment with the small molecule ISRIB to bypass the effects of PERK-dependent phosphorylation of the translation initiation factor eIF2α had no such effect, suggesting that cell cycle timing depends on PERK's kinase activity but is independent of eIF2α phosphorylation."

Finally, it is expected that PERK activation might be quickly turned off or neutralized by eIF2□ _phosphatase induction, as reported by several groups. Over a long time course (even 9 hours later), it might be challenging to observe, but this does not mean that it is not involved.

We agree, and therefore, we subjected cells to prolonged exposure to ISRIB (7 days), with daily drug replenishment. ISRIB is a potent ISR inhibitor (EC50 of 5nM) that renders cells insensitive to the effects of eIF2α phosphorylation, including GADD34 feedback control (PMID: 31402619). While our data—and interpretation of our results—do not rule out a potential effect of ISR feedback control in our experiments, any transient phosphorylation of eIF2α would be counteracted by ISRIB, and therefore, a major contribution of ISR feedback control remains unlikely.

Overall, experiments in Figure 1 and 2 may lay the foundation for developing a story with significant and concrete conclusions. However, the current resubmitted manuscript, despite limited editorial changes, fails to provide a sufficient advancement in our understanding of the UPR's role in the cell cycle and does not meet the caliber of papers typically published in *MBoC*.

We hope that these revisions to our manuscript are sufficient to clarify the concerns that were noted. We thank the reviewer for their time.

RE: Manuscript #E23-11-0419RRR
TITLE: "Baseline Unfolded Protein Response Signaling Adjusts the Timing of the Mammalian Cell Cycle"

Dear Dr. Zappa:

I am pleased to accept your manuscript for publication in Molecular Biology of the Cell.

Thank you for revising your Significance Statement according to MBoC guidelines. We are pleased to publish your manuscript in MBoC. Congratulations to you and your colleagues on an elegant and important study.

Sincerely,
Guillaume Thibault
Monitoring Editor
Molecular Biology of the Cell

Dear Dr. Zappa:

Congratulations on the acceptance of your manuscript! Thank you for choosing to publish your work in Molecular Biology of the Cell (MBoC).

Within 10 days, an unedited PDF of your manuscript will be published on MBoC in Press, an early release version of the journal. The date your manuscript appears on this site is the official publication date.

Your copyedited and typeset manuscript will be scheduled for publication in the next available issue of MBoC. Our production team will notify you when the page proofs of your paper are ready for your review.

MBoC offers the option to publish your paper with immediate open access. Open access can increase the discoverability and usability of your research. If you would like to publish your paper with immediate open access but did not select this option during initial submission, please contact the MBoC Editorial Office (mbc@ascb.org).

We invite you to submit images related to your accepted manuscript to be considered for the journal cover.
Cover images must contain color, TIF Format
Size image at 7.4" wide x 5.85" deep
RGB and CMYK color (please submit two separate files, one in each format)
300 dpi resolution

Please also send a 1-paragraph description of the image that a general audience could understand. Include with the caption the name and institution of the individual(s) the image should be attributed to. Please provide this description in a Word file. You can send the file via email or by your favorite file transfer service to mboc@ascb.org.

Authors of Articles and Brief Communications are also encouraged to create a short video abstract to accompany their article when it is published. These video abstracts, known as Science Sketches, are up to 2 minutes long and will be published on YouTube and then embedded in the article abstract. Science Sketch Editors on the MBoC Editorial Board will guide you as you prepare your video. Information about how to prepare and submit a video abstract is available at www.molbiolcell.org/science-sketches. Please contact mboc@ascb.org if you are interested in creating a Science Sketch.

We look forward to publishing your paper in MBoC.
Sign up for MBoC's new content alerts here https://tinyurl.com/3bcsyk3e

Sincerely,

Eric Baker
Journal Production Manager
MBoC Editorial Office
mbc@ascb.org
